# Detection of colonic dysplasia in patients with ulcerative colitis using a targeted fluorescent peptide and confocal laser endomicroscopy: A pilot study

**DOI:** 10.1371/journal.pone.0180509

**Published:** 2017-06-30

**Authors:** Giovanni Domenico De Palma, Irene Colavita, Gerardo Zambrano, Mariano Cesare Giglio, Francesco Maione, Gaetano Luglio, Giovanni Sarnelli, Antonio Rispo, Pietro Schettino, Francesco Paolo D’Armiento, Fatima Domenica Elisa De Palma, Valeria D’Argenio, Francesco Salvatore

**Affiliations:** 1 Department of Clinical Medicine and Surgery, University of Naples Federico II, Naples, Italy; 2 CEINGE-Biotecnologie Avanzate, Naples, Italy; 3 Department of Molecular Medicine and Medical Biotechnologies, University of Naples Federico II, Naples, Italy; 4 Department of Advanced Biomedical Sciences, University of Naples Federico II, Naples, Italy; Istituto di Ricovero e Cura a Carattere Scientifico Centro di Riferimento Oncologico della Basilicata, ITALY

## Abstract

**Aim:**

Targeted molecular probes have been used to detect sporadic colonic dysplasia during confocal laser endomicroscopy (CLE) with promising results. This is a feasibility pilot study aiming to assess the potential role of CLE combined with a fluorescent-labeled peptide to stain and detect dysplasia associated with Ulcerative Colitis.

**Method:**

A phage-derived heptapeptide with predicted high binding affinity for dysplastic tissue, was synthesized and labeled with fluorescein. Eleven lesions with suspected dysplasia at endoscopy were excised from nine patients with long-standing ulcerative colitis. Specimens were sprayed with the peptide and examined by CLE. The CLE images were then compared to the corresponding histological sections.

**Results:**

At definitive histology, 4 lesions were diagnosed as inflammatory polyps, 6 as dysplastic lesions and one as invasive cancer. In inflammatory polyps, the fluorescence signal came from peri-cryptal spaces and crypt lumen due to passive accumulation of the peptide in these areas. Dysplasia was associated with active binding of the peptide to dysplastic colonocytes.

**Conclusion:**

*Ex vivo* staining of ulcerative colitis-associated dysplasia using a fluorescent labeled molecular probe and CLE is feasible. *In vivo* studies on larger populations are required to evaluate the safety and the effective contribution of molecular probes in cancer surveillance of ulcerative colitis.

## Introduction

Patients with long-standing ulcerative colitis (UC) have an increased risk of developing colorectal cancer (CRC). This neoplasia has a cumulative incidence of 2%, 8% and 18% after 10, 20 and 30 years of disease, respectively [1]. Patients affected by long-standing UC are therefore candidates for surveillance colonoscopy to detect dysplasia [2,3]. Other than in association with polypoid lesions, dysplasia in UC can also develop in non-polypoid lesions or even within unremarkable mucosa (invisible dysplasia) [4]. Therefore, enhanced optical imaging techniques have been used to facilitate detection of dysplasia in these patients.

Chromo-endoscopy with indigo carmine or methylene blue has been particularly successful in this setting. Indeed, this technique increases the probability of detecting dysplasia by 3 to 5-fold compared to random biopsies [5,6], and is now considered a crucial tool in the surveillance of patients with inflammatory bowel diseases (IBD) [7].

Another contribution to this field was confocal laser endomicroscopy (CLE). This method provides *in vivo* high-magnified images of microscopic details of the gastrointestinal mucosa thereby enabling a diagnosis of early CR C and detection of dysplasia in UC patients [8,9]. Chromo-guided CLE has a high accuracy (97.8%) in the diagnosis of neoplastic changes in these patients [10]. Recent years have seen a growing interest in the combined use of targeted probes and new imaging technologies for early detection of cancer. Fluorescein-labeled probes, such as antibodies against neoplastic antigens [11–14] or phage-derived peptides binding dysplastic tissue [15,16] have been used in combination with CLE [17]. These latter probes are perhaps the most promising thanks to their low cost and limited immunogenicity, other than the possibility of binding pre-malignant tissue [17]. *In-vivo* topical application of peptides has been demonstrated to be safe, resulting in accurate staining of adenomatous colonic dysplasia [15]. Consequently, these probes are promising candidates for the detection of dysplasia in surveillance programmes in UC.

This is a feasibility pilot study aiming to investigate the potential role of CLE combined with a fluorescent-labeled molecular probe to stain and detect dysplasia in patients with long-standing UC.

## Methods

### Peptide synthesis and purification

The VRPMPLQ peptide [15] was synthesized on a BiotageSyroWave instrument, a system that combines conventional room temperature and microwaves peptide synthesis, at 25 μmol scale. Fluorescein conjugation was performed at the peptide N-terminus, via an aminohexanoic acid linker, using standard9-fluorenylmethoxycarbonyl (Fmoc) chemistry protocols. Fmocdeprotection was carried out with 25% piperidine in DMF for 5 +10 min at 25°C.Solutions of Fmoc-AA, HOBT/HBTU, and DIPEA were prepared at concentrations of 0.5M, 0.45M, and 1M in DMF, respectively. Coupling was performed with a 5-fold excess of Fmoc-AA-OH with 1:1:2 AA/HOBT-HBTU/DIPEA for 30 min at 25°C. Cleavage was carried out with 94:1:2.5:2.5 TFA/H_2_O/TIS/Thioanisole for 3 hours at 25°C, followed by precipitation and washings in tert-butyl methyl ether. Peptide analysis and purification were performed with a combined HPLC-ESI+-MS system (high performance liquid chromatography(Gilson, Middleton, WI, USA) coupled with a positiveelectrospray mass spectrometry ionization instrument (FlexarSQ 300 ESI+-MS, Perkin Elmer, Waltham, MA, USA). Analytical RP-HPLC made use of a Jupiter Proteo 10μm C18 250x4.6 mm column (Phenomenex, Torrance, CA, USA), monitoring at 220 and 280 nm, flow rate 1.3 mL/min, and the solvent system H_2_O/0.1% TFA (A) and CH_3_CN/0.1% TFA (B), with a linear gradient 5–60% B over 30 min. Peptide purification was performed by semi-preparative RP-HPLC on a Jupiter Proteo 90 Å C18 column (10x250 mm, 10 μm) (Phenomenex, Torrance, CA, USA), using a linear gradient 5–60% B over 25 min, with a flow rate of 10 mL/min. The identity (peptide molecular weight 1311.8) and the peptide purity (98%) were assessed by HPLC-ESI+-MS analysis. MS scan was performed in the mass range 200–3000 m/z. The raw materials used for peptide synthesis and purification are listed in *Supporting Infromation, S1 Appendix 1.* Lastly, a100 μM solution of VRPMPLQ peptide was prepared.

### Patients

Between March 2015 and March 2016, patients undergoing surveillance colonoscopy for long-standing UC and presenting endoscopically resectable lesions within colitis were considered for enrollment. The presence of quiescent UC at endoscopy (Mayo Ulcerative Colitis Endoscopic Score of Severity) [18] was required for inclusion.

### Endoscopy

All patients underwent colonoscopy preparation using a polyethylene glycol-based solution. Conscious sedation was obtained by administration of intravenous midazolam. Endoscopic examinations consisted of ileo-colonoscopies and were performed usingOlympus CF-HQ190 or CF TYPE Q180AL/I colonoscopes. After cecal intubation, the colonoscope was slowly withdrawn and the mucosa carefully inspected for endoscopically visible lesions. Lesions were classified according to the ASGE Standards of Practice Committee Guideline for the role of endoscopy in inflammatory bowel disease [19]. Lesions suspicious for dysplasia were evaluated for endoscopic resectability. Polypoid and non-polypoid lesions with distinct borders and without endoscopic features of submucosal invasion were removed en-bloc by endoscopic mucosal resection. Biopsies were obtained from flat and normal appearing mucosa surrounding the resection site.

### Ex-vivo tissue imaging

Specimens were rinsed with water and then sprayed with the 100μVRPMPLQ peptide solution within 15 min after excision. After an incubation time of 10 minutes [15], specimens were rinsed again to remove the excess of peptide not specifically bound to the tissue. CLE examination was then performed, moving the probe close to the mucosal surface of the specimen. The examination was carried-out with the Cellvizio® Endomicroscopy System (Mauna Kea Technologies, Paris, France), which uses a 2.5-mm catheter probe (Coloflex UHD-type probe). This system has a field of view of 240 x 200 μm, with a lateral resolution of 1 μm. CLE images were collected at a scan rate of 12 frames per second with a scanning field of 30,000 pixels.

At the end of the CLE examination, specimens were fixed in 10% formalin and processed for routine histological examination. Each specimen was classified according to the site of excision and was then assigned a unique number to enable the matching and comparison of histological hematoxylin-eosin sections with the corresponding CLE images.

Mosaic reconstructions were carried out using the Cellvizio® Viewer software, version 1.6.2 (Mauna Kea Technologies, Paris, France).

### Ethical considerations

Patients were excluded in case of age <18 years, refusal to participate or inability to provide written informed consent, which was obtained from all participants. The study protocol conforms to the ethical guidelines of the 1975 Declaration of Helsinki (6th revision, 2008) andhas been approved by the “Federico II” University of Naples Institutional Review board–Comitato Etico “C. Romano” (Protocol N.132/2015).

## Results

Nine patients were enrolled in the study. All patients had long-standing UC (> 10 years) and presented quiescent UC [18] at surveillance colonoscopy. A total of 11 lesions were obtained from the enrolled patients. In particular, 7 polypoid lesions and 4 non-polypoid were detected at endoscopy and eventually resected. At definitive histology, 4 lesions were diagnosed as inflammatory psudo-polyps, 6 as dysplastic lesions (low-grade dysplasia) and one as invasive cancer (SM3). Table 1 shows the histological characteristics of the collected lesions.

**Table 1:** Characteristics of excised lesions as resulted after histological evaluations. For Patient 3 and 7, two different lesions were collected and analyzed.

Patient	Type of lesion	Site	Histology
1	Polypoid	Sigmoid colon	Dysplasia
2	Polypoid	Transverse colon	Dysplasia
3	Polypoid Non-polypoid	Left colon Left colon	Dysplasia Dysplasia
4	Polypoid	Right colon	Inflammatory pseudo polyp
	Non-polypoid	Sigmoid colon	Inflammatory pseudo polyp
6	Polypoid	Recto sigmoid junction	Invasive Cancer
7	Polypoid Non-polypoid	Left colon Left colon	Dysplasia Inflammatory pseudo polyp
8	Polypoid	Left colon	Dysplasia
9	Non-polypoid	Sigmoid colon	Inflammatory pseudo polyp

### *Ex-vivo* VRPMPLQ-enhanced CLE

**Fig 1:**
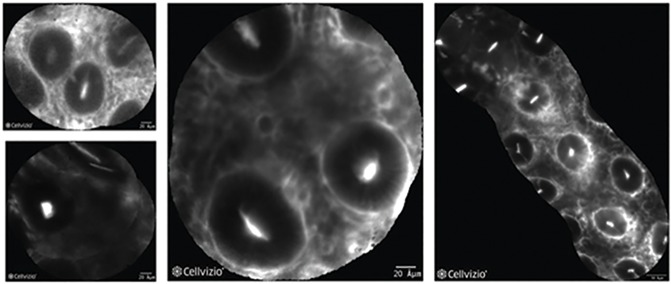
Non-dysplastic mucosa at CLE with heptapeptide (VRPMPLQ). A series of VRPMPLQ/CLE images from different patients showing non-dysplastic colonocytes. The fluorescence signal is seen emanating from areas corresponding to the pericryptal spaces and, to a much lesser extent, from the crypts-lumen. Crypts are highlighted in negative.

In non-dysplastic mucosa, the fluorescence signal was observed in areas corresponding to peri-cryptal spaces. Crypts were therefore highlighted in negative (Fig 1).

**Fig 2:**
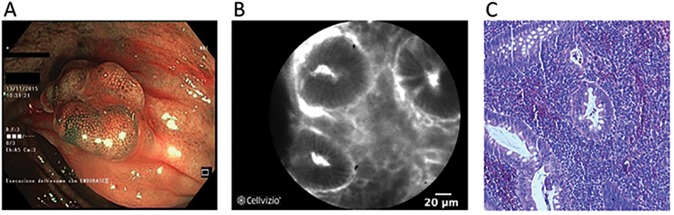
Non-dysplastic lesion. NBI-endoscopic view showing a polypoid lesion (Is, Paris classification) of the right colon (A). After resection and coloration with the 100μ VRPMPLQ peptide solution, CLE shows accumulation of the fluorescent peptide in the enlarged inter-crypt spaces and in the lumen crypts. Crypts are therefore highlighted in negative (B). Conventional histology (haematoxylin/eosin, original magnification, X 106) showing inflammatory psudopolyp (C).

In case of inflammatory polyps, CLE findings of the lesion together with the flat surrounding tissue were similar to non-dysplastic mucosa, showing passive accumulation of the fluorescent peptide in the enlarged inter-crypt spaces and in the lumen of the crypts (Fig 2).

**Fig 3:**
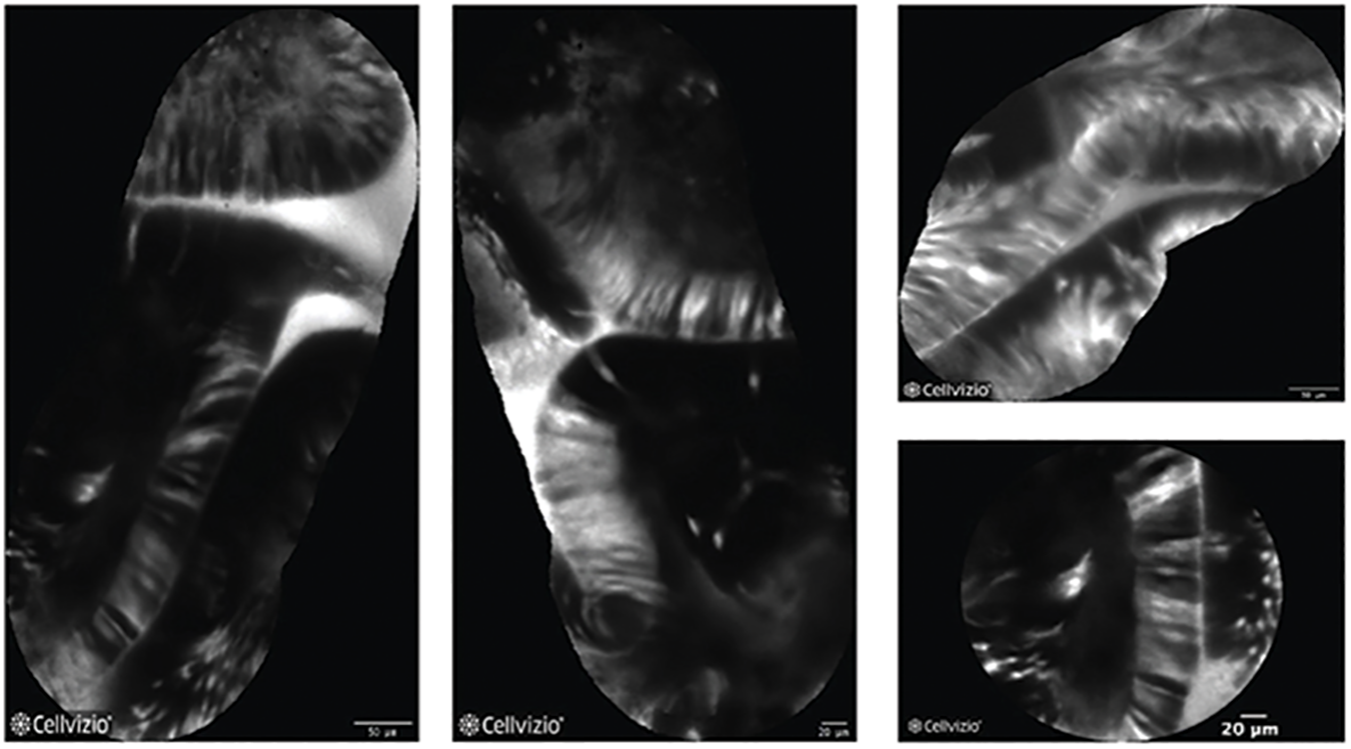
Dysplastic mucosa at CLE with heptapeptide (VRPMPLQ). A series of VRPMPLQ/CLE images from different patients showing dysplastic colonocytes.The active binding of the peptide to the colonocytes is observed and determinates a strong increase in fluorescence.

**Fig 4:**
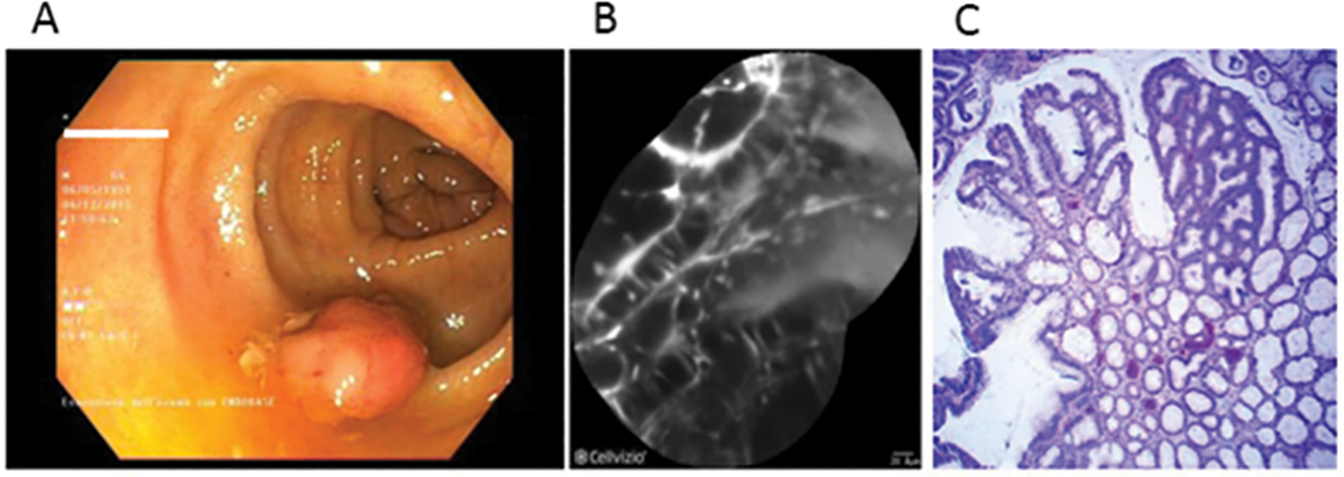
Dysplastic lesion. White-light endoscopic view showing a polypoid lesion (Is, Paris classification) of the transverse colon (A). After resection and coloration with the 100μ VRPMPLQ peptide solution, CLE shows active binding of the peptide to dysplastic colonocytes is observed. This along with passive accumulation of the peptide determines an increase in fluorescence (B). Conventional histology (haematoxylin/eosin, original magnification, X 106) showing low-grade dysplasia (C).

On the other hand, in presence of dysplasia, active binding of the peptide to dysplastic colonocytes was observed (Fig 3, Fig 4).

## Discussion

Several studies have assessed the feasibility of detecting colonic dysplasia using targeted fluorescent probes [17]. Antibodies against specific antigens or peptides showing binding affinity for dysplastic tissue have been tested in animal models of CRC [11,20] and in patients with sporadic adenomas or CRC [13,15]. In particular, *in vivo* studies have yielded encouraging results in terms of sensitivity and specificity [15]. In this promising context, the possibility of detecting neoplastic changes associated to IBD using molecular CLE has remained unexplored to date. To our knowledge, this is the first study to assess and confirm the possibility to detect UC-associated dysplasia at ex-vivo CLE examination using a molecular staining probe. In particular, we used the fluorescein-conjugated VRPMPLQ peptide as a molecular probe. This is a phage-derived heptapeptide that has been reported to highlight *sporadic* colonic dysplasia *in vivo* during CLE.^15^In this study, for the first time, the VRPMPLQ peptide has demonstrated to be able to detect also the dysplasia associated with Ulcerative Colitis. We believe this result is far from being obvious. Indeed, VRPMPLQ was originally selected because of its binding affinity to sporadic adenomatous polyps [15]. Like most sporadic cancers, sporadic adenomatous polyps are usually sustained by a chromosomal instabilitypathway [21],while neoplastic changes in UC are more frequently associated to the microsatellite instability pathway [22]. These different genetic pathways result in different phenotypes [23] that might result in different affinity for this probe, due to differential expression of the molecular target of the VRPMPLQ peptide, that is unknown yet [15].

In the present study the specificity of the probe for dysplasia areas was corroborated by the different distribution of the peptide observed depending on the absence or presence of dysplasia itself, as determined by traditional histology. In absence of dysplasia, a passive accumulation of the peptide in the lumen crypts and peri-cryptal spaces was observed, as previously described [15]. Thanks to this passive distribution pattern, the tissue architecture (i.e. crypts) is highlighted in negative contrast. Conversely, in the presence of dysplasia, active binding of the peptide to the colonocytes was observed. These findings open new scenarios in the management of lesions discovered in the context of UC. In presence of an endoscopically resectable lesion, topic application of the fluorescent VRPMPLQ peptide could allow a precise definition of the lesion borders, avoiding positive resection margins. Certainly, this theoretical advantage need to be proved by comparative studies on a larger number of lesions. Furthermore, it would allow a detailed examination of the flat mucosa surrounding the lesion, looking for further dysplasia to be discovered, thus orienting biopsies and avoiding unnecessary resections, when not indicated. In addition, topical administration of this molecular contrast avoids intravenous administration of fluorescein, thereby avoiding its adverse systemic effects, and enabling CLE in patients in whom administration of systemic fluorescein is contraindicated [24,25].

Further studies on larger population of UC patients are needed to estimate the accuracy and the additional value of targeted CLE compared to chromoendoscopy alone, also in terms of cost/benefits. Indeed, the use of CLE in cancer surveillance remains restricted to suspicious areas highlighted by methylene blue or indigo carmine, because examination of the whole colonic mucosa by CLE remains impracticable [7]. Wanders and colleagues found that the combination of chromoendoscopy and CLE has limited applicability in the surveillance of IBD patients due to a low sensitivity (0.43) [26]. However, their study was hindered by the frequent technique failure of the endoscope-integrated system of CLE (iCLE) used by the authors. The iCLE system integrates the CLE in the tip of the endoscope, in contrast with the probe-based CLE (pCLE), used in this study, where a CLE probe is advanced through the working channel of a standard endoscope. On the contrary, Duglosz and colleagues found good accuracy of pCLE in differentiating neoplastic from non-neoplastic mucosa during surveillance in patients with IBD with high sensitivity (0.89) [26]. A major drawback of the CLE investigation of inflamed mucosa is the risk of false positives for dysplasia due to inflammatory changes as crypt destruction and fluorescein leakage, which can be misdiagnosed as dysplasia [27,28]. Hence, molecular CLE could improve the specificity of classical CLE in the presence of inflammation thanks to the specificity of the binding probe-dysplasia.

There are several limitations to this study, although most of them are intrinsic to feasibility studies. First, this is a pilot study limited to an *ex-vivo* investigation, as required by the local ethics committee. Given the experience of Hsiung et al. [15], we are confident that topical administration *in-vivo* of the peptide would highlight dysplastic colonocytes in UC patients as well. Nevertheless, this will be the next step of our research. Second, a control peptide was not used to test the specificity of the binding. Indeed, we relied on the observation of Hsiung et al. [15] and the peptide also showed a different pattern of distribution on non-dysplastic lesions. Third, the study was limited to UC patients with quiescent disease. Indeed, the presence of an inflamed mucosa could affect the peptide binding. Increased mucosal permeability and inter-crypt distance might favour passive accumulation of the peptide (i.e., non-specific binding), making it difficult to differentiate between active and passive binding. In addition, safety issues might emerge in case of *in-vivo* administration in patients with active disease. Although peptides have been preferred to antibodies in view of their reduced immunogenicity [17], the quota of systemic absorption might increase in the presence of altered mucosa. Lastly, we tested the peptide on a small population of lesions in UC patients. These lesions might not account for the entire variety of lesions in terms of genotype and phenotype that might be found in UC patients.

In conclusion, the VRPMPLQ peptide is a promising probe for the detection of dysplasia in the context of IBD. Further *in vivo* studies on larger populations are required to evaluate the effective contribution of this molecular probe in the management of lesions found during surveillance of UC patients.

## Supporting information

S1 Appendix Raw materials used for peptide synthesis and purification. (DOCX)

## References

[1] Munkholm P. Review article: the incidence and prevalence of colorectal cancer in inflammatory bowel disease. Aliment Pharmacol Ther 2003;18 Suppl 2:1–5. 10.1046/j.1365-2036.18.s2.2.x 12950413

[2] Van Assche G, Dignass A, Bokemeyer B, Danese S, Gionchetti P, Moser G, et al Second European evidence-based consensus on the diagnosis and management of ulcerative colitis part 3: special situations. J Crohns Colitis 2013;7:1–33. doi: 10.1016/j.crohns.2012.09.005 23040453 10.1016/j.crohns.2012.09.005

[3] Farraye FA, Odze RD, Eaden J, Itzkowitz SH.AGA technical review on the diagnosis and management of colorectal neoplasia in inflammatory bowel disease. Gastroenterology, 2010;138:746–74. doi: 10.1053/j.gastro.2009.12.035 20141809 10.1053/j.gastro.2009.12.035

[4] Broström O, Löfberg R, Ost A, Reichard H. Cancer surveillance of patients with longstanding ulcerative colitis: a clinical, endoscopical, and histological study. Gut 1986;27:1408–13. 3804019 10.1136/gut.27.12.1408 PMC1433971

[5] Hurlstone DP, Sanders DS, McAlindon ME, Thomson M, Cross SS. High-magnification chromoscopic colonoscopy in ulcerative colitis: a valid tool for in vivo optical biopsy and assessment of disease extent. Endoscopy 2006;38:1213–7. doi: 10.1055/s-2006-944732 17163321 10.1055/s-2006-944732

[6] Kiesslich R, Fritsch J, Holtmann M, Koehler HH, Stolte M, Kanzler S, et al Methylene blue-aided chromoendoscopy for the detection of intraepithelial neoplasia and colon cancer in ulcerative colitis. Gastroenterology 2003;124:880–8. doi: 10.1053/gast.2003.50146 12671882 10.1053/gast.2003.50146

[7] Laine L, Kaltenbach T, Barkun A, McQuaid KR, Subramanian V, Soetikno R. SCENIC international consensus statement on surveillance and management of dysplasia in inflammatory bowel disease. Gastrointest Endosc 2015;81:489–501. doi: 10.1016/j.gie.2014.12.009 25708752 10.1016/j.gie.2014.12.009

[8] De Palma GD, Staibano S, Siciliano S, Persico M, Masone S, Maione F, et al In vivo characterisation of superficial colorectal neoplastic lesions with high-resolution probe-based confocal laser endomicroscopy in combination with video-mosaicing: a feasibility study to enhance routine endoscopy. Dig Liver Dis 2010;42:791–7. doi: 10.1016/j.dld.2010.03.009 20409761 10.1016/j.dld.2010.03.009

[9] Rispo A, Castiglione F, Staibano S, Esposito D, Maione F, Siano M, et al Diagnostic accuracy of confocal laser endomicroscopy in diagnosing dysplasia in patients affected by long-standing ulcerative colitis. World J Gastrointest Endosc 2012;4:414–20. doi: 10.4253/wjge.v4.i9.414 23125900 10.4253/wjge.v4.i9.414 PMC3487190

[10] Kiesslich R, Goetz M, Lammersdorf K, Schneider C, Burg J, Stolte M, et al Chromoscopy-guided endomicroscopy increases the diagnostic yield of intraepithelial neoplasia in ulcerative colitis. Gastroenterology 2007;132:874–82. doi: 10.1053/j.gastro.2007.01.048 17383417 10.1053/j.gastro.2007.01.048

[11] Foersch S, Kiesslich R, Waldner MJ, Delaney P, Galle PR, Neurath MF, et al Molecular imaging of VEGF in gastrointestinal cancer in vivo using confocal laser endomicroscopy. Gut 2010;59:1046–55. doi: 10.1136/gut.2009.202986 20639250 10.1136/gut.2009.202986

[12] Goetz M, Ziebart A, Foersch S, Vieth M, Waldner MJ, Delaney P, et al In vivo molecular imaging of colorectal cancer with confocal endomicroscopy by targeting epidermal growth factor receptor. Gastroenterology 2010;138:435–46. doi: 10.1053/j.gastro.2009.10.032 19852961 10.1053/j.gastro.2009.10.032

[13] Liu J, Zuo X, Li C, Yu T, Gu X, Zhou C, et al In vivo molecular imaging of epidermal growth factor receptor in patients with colorectal neoplasia using confocal laser endomicroscopy. Cancer Lett 2013;330:200–7. doi: 10.1016/j.canlet.2012.11.044 23220286 10.1016/j.canlet.2012.11.044

[14] Cârţână T, Săftoiu A, Gruionu LG, Gheonea DI, Pirici D, Georgescu CV, et al Confocal laser endomicroscopy for the morphometric evaluation of microvessels in human colorectal cancer using targeted anti-CD31 antibodies. PLoS One 2012;7:e52815 doi: 10.1371/journal.pone.0052815 23285192 10.1371/journal.pone.0052815 PMC3532115

[15] Hsiung P-L, Hsiung P-L, Hardy J, Friedland S, Soetikno R, Du CB, et al Detection of colonic dysplasia in vivo using a targeted hheptapeptide and confocal microendoscopy. Nat Med 2008;14:454–8. doi: 10.1038/nm1692 18345013 10.1038/nm1692 PMC3324975

[16] Sturm MB, Joshi BP, Lu S, Piraka C, Khondee S, Elmunzer BJ, et al Targeted imaging of esophageal neoplasia with a fluorescently labeled peptide: first-in-human results. Sci Transl Med 2013;5:184ra61 doi: 10.1126/scitranslmed.3004733 23658246 10.1126/scitranslmed.3004733 PMC3859345

[17] Karstensen JG, Klausen PH, Saftoiu A, Vilmann P. Molecular confocal laser endomicroscopy: a novel technique for in vivo cellular characterization of gastrointestinal lesions. World J Gastroenterol 2014;20:7794–800. doi: 10.3748/wjg.v20.i24.7794 24976717 10.3748/wjg.v20.i24.7794 PMC4069308

[18] Rutgeerts P, Sandborn WJ, Feagan BG, Reinisch W, Olson A, Johanns J, et al Infliximab for induction and maintenance therapy for ulcerative colitis. N Engl J Med 2005;353:2462–76. doi: 10.1056/NEJMoa050516 16339095 10.1056/NEJMoa050516

[19] ASGE Standards of Practice Committee, Shergill AK, Lightdale JR, Bruining DH, Acosta RD, Chandrasekhara V, et al ASGE guideline: The role of endoscopy in inflammatory bowel disease. Gastrointest Endosc. 2015 5;81(5):1101–21.e1-13. doi: 10.1016/j.gie.2014.10.030 25800660 10.1016/j.gie.2014.10.030

[20] Goetz M, Hoetker MS, Diken M, Galle PR, Kiesslich R. In vivo molecular imaging with cetuximab, an anti-EGFR antibody, for prediction of response in xenograft models of human colorectal cancer. Endoscopy 2013;45:469–77. doi: 10.1055/s-0032-1326361 23580409 10.1055/s-0032-1326361

[21] Pino MS, Chung DC. The chromosomal instability pathway in colon cancer. Gastroenterology 2010;138:2059–72. doi: 10.1053/j.gastro.2009.12.065 20420946 10.1053/j.gastro.2009.12.065 PMC4243705

[22] Fujiwara I, Yashiro M, Kubo N, Maeda K, Hirakawa K. Ulcerative colitis-associated colorectal cancer is frequently associated with the microsatellite instability pathway. Dis Colon Rectum 2008;51:1387–94. doi: 10.1007/s10350-008-9212-9 18546042 10.1007/s10350-008-9212-9

[23] Dunican DS, McWilliam P, Tighe O, Parle-McDermott A, Croke DT. Gene expression differences between the microsatellite instability (MIN) and chromosomal instability (CIN) phenotypes in colorectal cancer revealed by high-density cDNA array hybridization. Oncogene 2002;21:3253–7. doi: 10.1038/sj.onc.1205431 12082642 10.1038/sj.onc.1205431

[24] Wallace MB, Meining A, Canto MI, Fockens P, Miehlke S, Roesch T. The safety of intravenous fluorescein for confocal laser endomicroscopy in the gastrointestinal tract. Aliment Pharmacol Ther 2010;31:548–52. doi: 10.1111/j.1365-2036.2009.04207.x 20002025 10.1111/j.1365-2036.2009.04207.x

[25] Paramsothy S, Leong RWL. Endoscopy: Fluorescein contrast in confocal laser endomicroscopy. Nat Rev Gastroenterol Hepatol 2010;7:366–8. doi: 10.1038/nrgastro.2010.83 20606632 10.1038/nrgastro.2010.83

[26] Wanders LK, Kuiper T, Kiesslich R, Karstensen JG, Leong RW, Dekker E, et al Limited applicability of chromoendoscopy-guided confocal laser endomiscroscopy as daily-practice surveillance strategy in Crohn’s disease. Gastrointest Endosc 2016;85:966–971. 10.1016/j.gie.2015.09.001 26358329

[27] Dlugosz A, Barakat AM, Björkström NK, Öst Å, Bergquist A. Diagnostic yield of endomicroscopy for dysplasia in primary sclerosing cholangitis associated inflammatory bowel disease: a feasibility study. Endosc Int Open. 2016;4:E901–11 doi: 10.1055/s-0042-111203 27540581 10.1055/s-0042-111203 PMC4988862

[28] Thorlacius H1, Toth E. Role of chromoendoscopy in colon cancer surveillance in inflammatory bowel disease. Inflamm Bowel Dis. 2007;13:911–917. doi: 10.1002/ibd.20118 17309075 10.1002/ibd.20118

