# Supplementary material for: Detection of colonic dysplasia in patients with ulcerative colitis using a targeted fluorescent peptide and confocal laser endomicroscopy: A pilot study
Source: PLoS One. 2017 Jun 30;12(6):e0180509. doi: 10.1371/journal.pone.0180509 (PMC5493408; doi:10.1371/journal.pone.0180509)
Supplement: S1 Appendix — (DOCX) [file pone.0180509.s001.docx]

**Supporting information. Appendix 1**

**Row materials used for peptide synthesis and purification**

Fmoc amino acids, Diisopropylethylamine (DIPEA), piperidine, Acetic anhydride, trifluoroacetic acid (TFA), triisopropylsilane (TIS), Dichloromethane (DCM), *N*,*N*-dimethylformamide (DMF), tert-butyl methyl ether, N,N’-dicycloexylcarbodiimide (DCC), HPLC grade water and acetonitrile (Sigma Aldrich, St. Louis, MO), 1-Hydroxybenzotriazole hydrate (HOBT) and O-(Benzotriazol-1-yl)-*N*,*N*,*N*’,*N*’-tetramethyluroniumhexafluorophosphate (HBTU), 5-carboxyfluorescein, succinimidyl ester and Fmoc-L aminocaproic acid(AnaSpec, Inc. Fremont, CA) and Rink-amide-MBHA resin (substitution 0.38 mmol/g) (Novabiochem San Diego, CA).
